# Educational Attainment Dynamics in Wales: Insights through Data Linkage and Geographically Weighted Regression

**DOI:** 10.23889/ijpds.v9i5.2722

**Published:** 2024-09-18

**Authors:** Alexandra Sandu, Jennifer Keating, Katy Huxley, Rob French, Anthony Whiffen

**Affiliations:** 1 Administrative Data Research Wales - Cardiff University; 2 Wales Institute of Social and Economic Research and Data - Cardiff University; 3 Spark|Sbarc - Cardiff University; 4 Welsh Government

Quantifying the determinants of educational attainment requires a multifaceted approach encompassing various domains, including individual, family, household, neighbourhood, and school. This study examines the importance of socio-economic status for educational attainment within Wales, shedding light on the ways this can be measured, the importance across domains, and how these relationships vary across geographic areas.

Two main research questions guide this analysis. Firstly, it asks, what is the correlation between educational attainment and a range of socio-economic indicators derived from linked data, including free school meals and household measures? Secondly, is there variability of these associations across different geographical levels within Wales?

Employing Geographically Weighted Regression, the study investigates the effects of spatial differences of socio-economic indicators’ on pupils' attainment. It achieves this by integrating multiple Wales-specific administrative datasets, such as pupil administrative records, school-level data, family/household demographics and deprivation measures through data linkage. This comprehensive approach provides a nuanced understanding and underscores the importance of integrated datasets in uncovering insights into pupils' attainment.

Our findings reveal disparities in how socio-economic factors and family/household characteristics are associated with pupils’ attainment across distinct geographies in Wales. These disparities are visually depicted through maps generated in ArcGIS Pro. This emphasis on geographical variations not only underscores the pivotal role of local contexts in understanding educational disparities but also highlights the complex interplay between these factors and pupils' attainment. Incorporating such visual representation enriches our analysis and provides a compelling rationale for targeted interventions to mitigate disparities in educational outcomes.

